# Altered Global Brain Functional Connectivity in Drug-Naive Patients With Obsessive-Compulsive Disorder

**DOI:** 10.3389/fpsyt.2020.00098

**Published:** 2020-03-03

**Authors:** Guangcheng Cui, Yangpan Ou, Yunhui Chen, Dan Lv, Cuicui Jia, Zhaoxi Zhong, Ru Yang, Yuhua Wang, Xin Meng, Hongsheng Cui, Chengchong Li, Zhenghai Sun, Xiaoping Wang, Wenbin Guo, Ping Li

**Affiliations:** ^1^ Department of Psychiatry, Qiqihar Medical University, Qiqihar, China; ^2^ Department of Psychiatry, The Second Xiangya Hospital of Central South University, Changsha, China; ^3^ Henan Key Lab of Biological Psychiatry, The Second Affiliated Hospital of Xinxiang Medical University, Xinxiang, China; ^4^ Department of Radiology, The Second Xiangya Hospital of Central South University, Changsha, China; ^5^ Department of Radiology, The Third Affiliated Hospital of Qiqihar Medical University, Qiqihar, China

**Keywords:** obsessive-compulsive disorder, global brain functional connectivity, functional magnetic resonance imaging, network, resting state

## Abstract

Abnormal functional connectivity (FC) within discrete brain networks is involved in the pathophysiology of obsessive-compulsive disorder (OCD) with inconsistent results. In the present study, we investigated the FC patterns of 40 drug-naive patients with OCD and 38 healthy controls (HCs) through an unbiased voxel-wise global brain FC (GFC) analysis at rest. Compared with HCs, patients with OCD showed decreased GFC within the default mode network (DMN) (i.e., left posterior cingulate cortex/lingual gyrus) and sensorimotor network (i.e., left precentral gyrus/postcentral gyrus) and increased GFC within the executive control network (ECN) (i.e., left dorsal lateral prefrontal cortex and left inferior parietal lobule). Receiver operating characteristic curve analyses further indicated that the altered GFC values within the DMN, ECN, and sensorimotor network may be used as neuroimaging markers to differentiate patients with OCD from HCs. These findings indicated the aberrant FC patterns of the DMN, ECN, and sensorimotor network associated with the pathophysiology of OCD and provided new insights into the changes in brain organization function in OCD.

## Introduction

Obsessive-compulsive disorder (OCD) is a common and chronic psychiatric disorder involving intrusive, unwanted thoughts, and/or repetitive behavior, anxiety, and social dysfunction. OCD has a lifetime prevalence of 2%–3% worldwide (1), but the underlying neurobiological mechanisms remain unclear.

Neuroimaging studies have shown that OCD may be caused by distortions within large-scale brain networks rather than from independent brain regions (2). A “triple network” model emphasizes the abnormal intrinsic functional connectivity (FC) patterns within and between the executive control network (ECN), salience network (SN), and default mode network (DMN) as essential features of psychiatric disorders including OCD (3). DMN is composed of posterior cingulate cortex (PCC), medial prefrontal cortex (PFC), and lateral posterior cortices, which is involved in reﬂective and introspective self-awareness processes (4). ECN is composed of dorsal lateral PFC (DLPFC) and posterior parietal cortex, which is crucial for cognitive control (such as planning and response inhibition) (5, 6). SN is composed of anterior insular cortices and dorsal anterior cingulate, which plays an important role in regulating external and internal salient information (3, 7). The DMN deactivates during cognitive demanding tasks and shows increased activities during resting state, and is considered as a task-negative network. The ECN shows elevated co-activation during the cognitive performance, and is a task-positive network. The SN is involved in monitoring the interactions between the DMN and ECN (8). FC within and between the DMN, ECN, and SN was reported to be altered in patients with OCD, and dysfunction of the triple networks may be related to obsessive thoughts and/or compulsive behavior in OCD (2).

Considerable neuroimaging studies have observed altered FC and/or regional activity in brain regions within the DMN, ECN, and SN with inconsistent results. Increased FC and/or regional homogeneity within the DMN, ECN, and SN at rest were found through using the independent component analysis and regions of interest (ROI) seed-based FC approaches (8–12). By contrast, neuroimaging studies have reported decreased FC within the DMN, ECN, and SN at rest in OCD (2, 13–15). Two main factors may explain the inconsistency of the findings. One factor may be the sample heterogeneity regarding medication use. Selective serotonin reuptake inhibitors (SSRIs) may modulate the neural condition and are known to have a remarkable inﬂuence on FC at rest in patients with OCD (16, 17). Therefore, drug-naive patients with OCD should be employed to obtain primitive FC information of the neurobiological mechanisms in OCD. Another important factor may be that numerous neuroimaging studies have concentrated on FC between brain regions of an appointed network rather than using a whole brain assessment (18). Thus, FC based on the assumptive network of interest may lead to biased results and limits the investigation of the most remarkably altered brain areas that may represent the essential abnormality in the neurobiological mechanisms of OCD (19). Voxel-wise global brain FC (GFC) analysis is a model-free method used to investigate functional interactions across the whole brain regions with an unbiased hypothesis-driven manner, and has been applied to obtain whole brain FC in patients with major depressive disorder (19), schizophrenia (20), and somatization disorder (21).

In this study, we compared GFC differences between drug-naive patients with OCD and healthy controls (HCs) using resting-state fMRI. On the basis of the aforementioned studies, we hypothesized that patients with OCD would show altered GFC in some brain regions, peculiarly in brain regions of the DMN, ECN, and SN. We also hypothesized that these alterations would be related to clinical variables in OCD.

## Materials and Methods

### Participants

Forty drug-naive patients with OCD were recruited from the Fourth Affiliated Hospital of Qiqihar Medical University and Qiqihar Mental Health Center. Patients with OCD were diagnosed using the Structured Clinical Interview for DMS-IV (SCID), patient version. Yale-Brown Obsessive-Compulsive Scale (Y-BOCS), 17-item Hamilton Rating Scale for Depression (HAMD), and Hamilton Anxiety Rating Scale (HAMA) were used to assess the severity of OCD, depression symptoms, and anxiety symptoms, respectively. Patients with OCD should have a score of higher than 16 on Y-BOCS and score of less than 18 on HAMD. In current OCD sample, 4 patients were with obsessions, 4 patients were with compulsion, and 32 patients were with both obsessions and compulsion. Forty HCs were recruited from the community and screened using the SCID, nonpatient version. The participants shared the following exclusion criteria: (1) diagnosis of other Axis I and II disorders; (2) a history of major physical diseases and neurological disorders; and (3) nicotine/caffeine dependence or substance/alcohol use disorder. Moreover, HCs with a family history of major psychiatric disorders were excluded. All participants were right-handed and aged from 18 to 60 years old.

This study was approved by the Research Ethics Committee at Qiqihar Medical University, China. All participants were informed regarding the procedures, and signed an informed consent.

### Image Acquisition and Preprocessing

Images were acquired by using a 3.0-Tesla GE 750 Signa-HDX scanner (General Electric Healthcare, Waukesha, WI, USA) at the Third Affiliated Hospital of Qiqihar Medical University, Heilongjiang, China. The subjects were instructed to relax, remain motionless (especially the head), keep eyes closed, and stay awake. An echo-planar imaging (EPI) sequence was used to acquire resting-state functional magnetic resonance imaging (RS-fMRI). The parameters were as follows: 33 axial slices, TR = 2,000 ms, TE = 30 ms, FA = 90°, thickness/gap = 3.5 mm/0.6 mm, FOV = 200 × 200 mm, and in-plane resolution = 64 × 64. A total of 240 volumes were collected for 8 min.

The images were manually check to ensure good coverage for all voxels included in a whole-braingray matter mask. Data Processing & Analysis for Brain Imaging (DPABI) software was used to preprocess the imaging data (22). After signal stabilization, slice timing determination and head-motion correction were conducted. The subjects included in the analysis should have a maximum displacement ≤ 2 mm in *x, y,* or *z* direction and an angular rotation < 2° on each axis. Two HCs were excluded from further analysis because of excessive head motion. The functional images were normalized to the standard EPI template in SPM8 (http://www.fil.ion.ucl.ac.uk/spm) and spatially resampled to a voxel size of 3 mm × 3 mm × 3 mm. The processed images were smoothed with a Gaussian kernel of 4 mm full width at the half maximum. The signal was linearly detrended and band-pass filtered (0.01–0.08 Hz) to reduce high-frequency physiological noise and low-frequency drift. The 24 head motion parameters obtained by rigid body correction, white matter, and cerebrospinal fluid time courses were removed from the images by linear regression. The global signal was included because removing it in the preprocessed RS-fMRI FC data remains debatable. Resting-state fMRI is highly susceptible to head motion and that simply regressing out the effects of the 24 head motion parameters is not sufficient to deal with this issue (23, 24). The analyses were therefore using scrubbing with a framewise displacement (FD) measure, which indexes volume-to-volume changes in head position using a threshold of 0.2 together with one preceding and two subsequent volumes (25).

### Global-Brain Functional Connectivity (GFC) Analysis

Voxel-wise GFC was calculated in MATLAB within a gray matter mask, which was created by thresholding (probability > 0.2) the gray matter probability map in SPM8 (26). GFC values for a given voxel of each participant were computed between this voxel and all other voxels within the gray matter mask. The formula used to calculate the GFC values was described by Cui et al. (19) as follows:

$$GFCa=\sum_{b=1}^{n}\frac{r(Ta,Tb)}{n-1}$$

In this formula, the symbols *a* and *b* stand for the given voxels *a* and *b*, *n* represents number, *r* means the Pearson' s correlation coefficient, *T_a_* and *T_b_* are the time series of voxel *a* and voxel *b*. The coefficient *r* was computed and transformed to *z* values with Fisher *r*-to-*z* transformation (27). GFC of a voxel was defined as the mean coefficient of the given voxel with all other voxels, and GFC maps were created by combining the GFC of all voxels.

### Statistical Analysis

Imaging data and demographics were compared between OCD and HCs. Two-sample t-tests were used to analyze the continuous variables, and chi-square tests were used to analyze the categorical data.

GFC analysis was conducted with two-sample t-tests between patients with OCD and HCs. The FD values of each participant were calculated based on the study of Power et al. (25) by indexing volume-to-volume changes in head position. The mean FD values and age were used as covariates to minimize the potential effects of these variables. The signiﬁcance level was set at the corrected *p* < 0.05 for multiple comparisons using the Gaussian Random Field (GRF) method (voxel significance: *p* < 0.001, cluster significance: *p* < 0.05).

Pearson and Spearman correlation analyses were used to assess the relationship between the GFC values showing signiﬁcant group differences (mean z values of GFC were extracted) and clinical variables in the patients with OCD. A partial correlation analysis was also calculated between the altered GFC values and clinical variables in OCD, the mean FD values and age were used as covariates. The significance level was Bonferroni corrected at *p* < 0.05.

Moreover, receiver-operating characteristic curve (ROC) analysis was conducted to determine whether the altered GFC values of these ROIs could be used to differentiate patients with OCD from HCs. A “leave-one-out” method was applied to conduct the ROC. Furthermore, a permutation test was used to validate the ROC results, which ran 10,000 times for each sample (OCD/HCs) and a global accuracy could be obtained for each sample.

## Results

### Characteristics of the Participants

**Supplementary Table S1** indicates the clinical characteristics of all participants. The patients had a mean age of 27.28 ± 8.16 years, a mean duration of illness of 66.68 ± 75.54 months, and a mean education of 13.40 ± 2.87 years. The controls had a mean age of 27.18 ± 8.33 years and a mean education of 13.74 ± 3.03 years. No significant difference was found regarding gender (*x^2^* = 0.32, *p* = 1.00), age (*t* = 0.05, *p* = 0.71), education (*t* = -0.50, *p* = 0.83), and FD values (*t* = 1.25, *p* = 0.13) between OCD and HC groups. However, significant group differences were found in Y-BOCS total (*t* = 25.27, *p < 0.001*) and subscale scores (obsessive thinking, *t* = 17.98, *p < 0.001;* compulsive behavior, *t* = 14.92, *p < 0.001*), HAMD scores (*t* = 9.04, *p < 0.001*), and HAMA scores (*t* = 9.00, *p < 0.001*) (**Supplementary Table S1**).

### Group Differences in GFC

**Figure 1:**
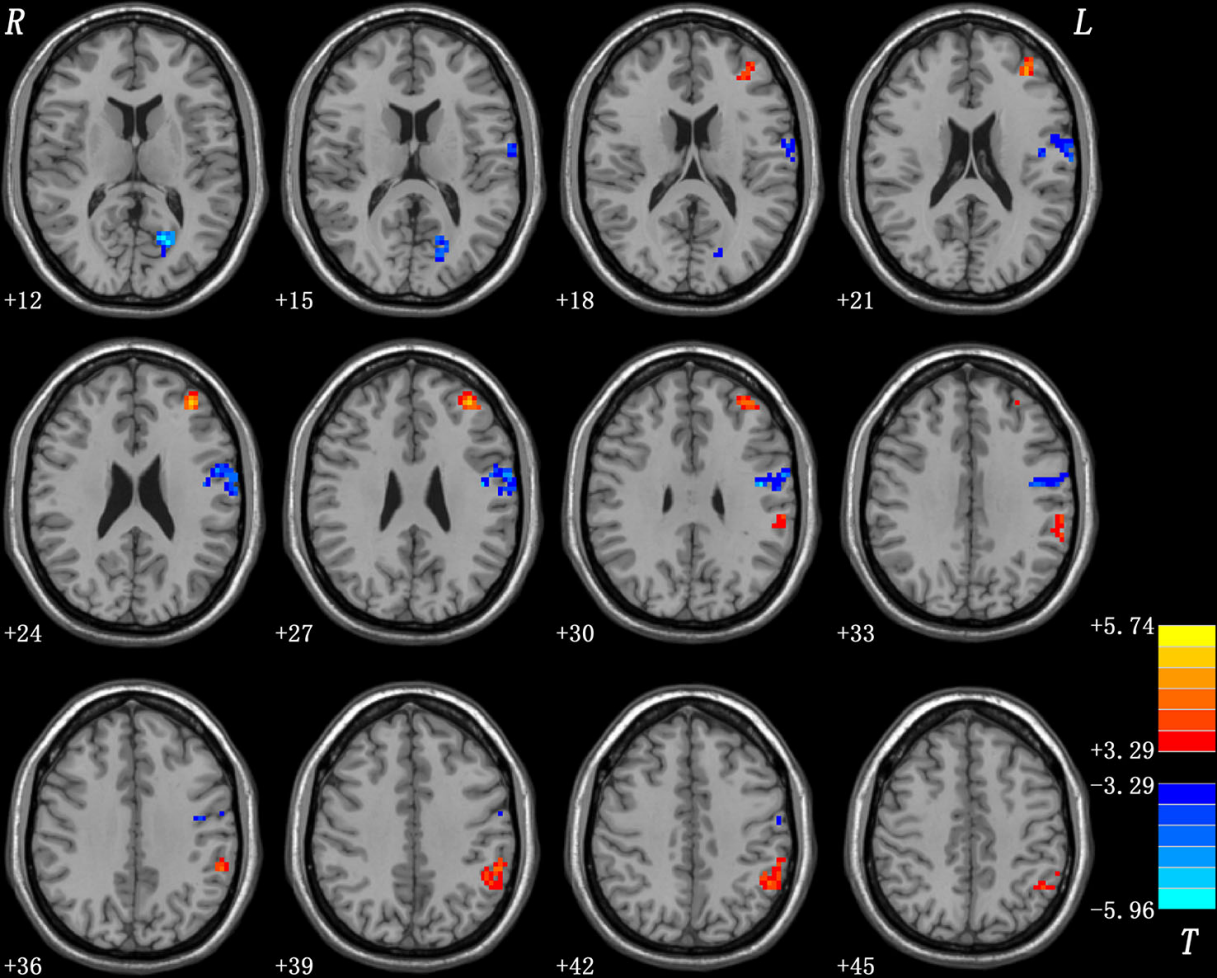
Brain regions with abnormal GFC in patients with OCD. The threshold was set at *p* < 0.05 corrected by the GRF method. Red and blue denote increased and decreased GFC values respectively. Colour bar indicates *t* values from two-sample *t*-tests. L, left side; R, right side; GFC, global-brain functional connectivity; OCD, obsessive-compulsive disorder; GRF, Gaussian Random Field.

**Figure 2:**
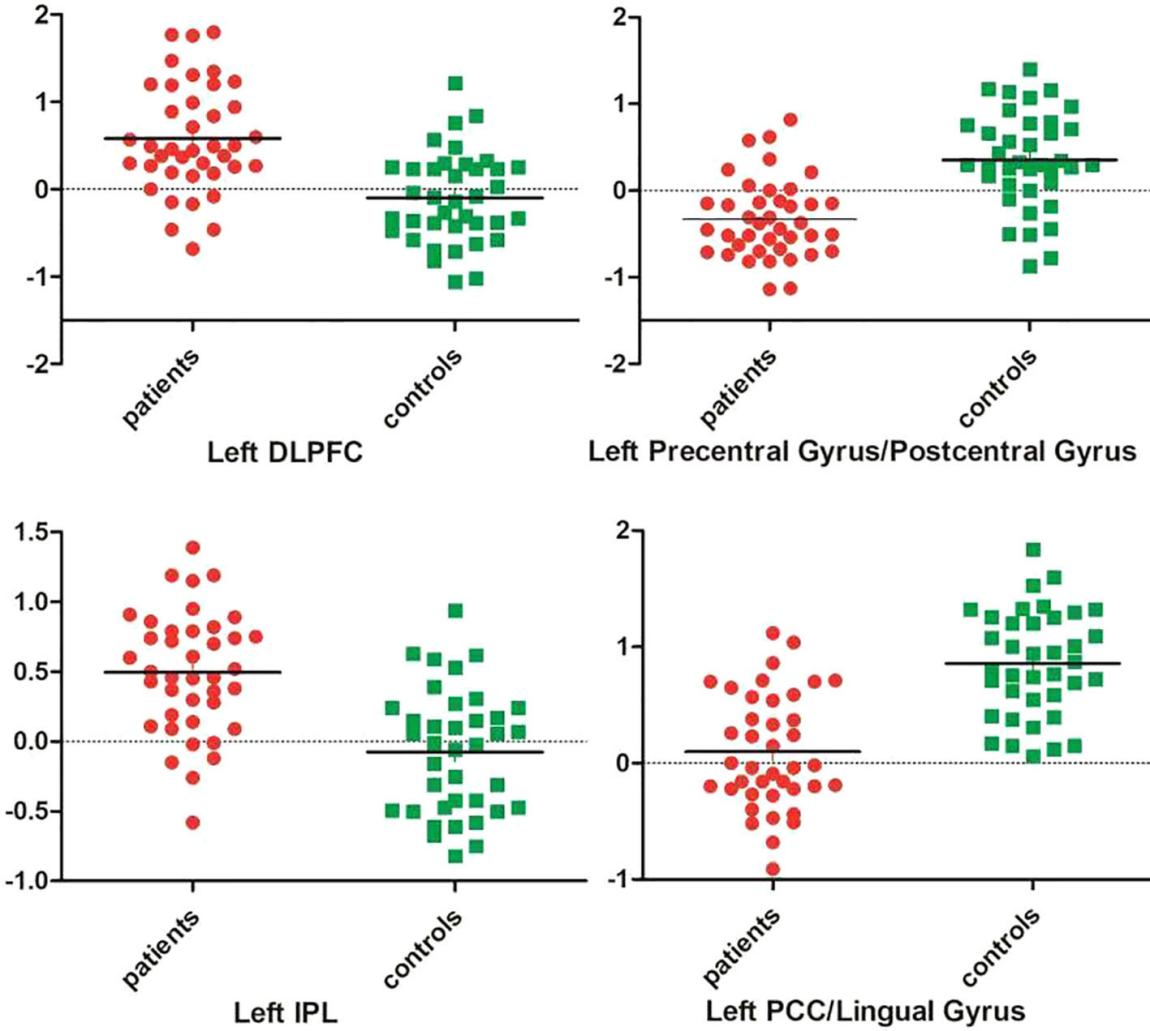
A plot of the extracted cluster values with the raw data points of samples. DLPFC, dorsal lateral prefrontal cortex; IPL, inferior parietal lobule; PCC, posterior cingulate cortex.

As shown in **Table 1** and **Figure 1**, patients with OCD showed significantly decreased GFC values in the left PCC/lingual gyrus and left precentral gyrus/postcentral gyrus and significantly increased GFC values in the left DLPFC and left inferior parietal lobule (IPL). A plot of the extracted cluster values with the raw data points of samples (OCD/HCs) was presented in **Figure 2**. A sagittal view of the left PCC/lingual gyrus cluster was presented in **Supplementary Figure S1**.

**Table 1:** Regions with abnormal GFC in the patients with OCD.

Cluster location	Peak (MNI)			Number of voxels	*T* value
	x	y	z		
Left PCC/Lingual Gyrus	−18	−66	12	83	−5.9634
Left Precentral Gyrus/Postcentral Gyrus	−45	−9	30	129	−5.6116
Left DLPFC	−36	45	27	56	5.2824
Left IPL	−54	−42	39	83	4.3242

GFC, global-brain functional connectivity; OCD, obsessive-compulsive disorder; MNI, Montreal Neurological Institute; PCC, posterior cingulate cortex; DLPFC, dorsal lateral prefrontal cortex; IPL, inferior parietal lobule.

There is a debate in whether global signal should be removed. We reanalyzed the data with global signal removal, and obtained the similar results (**Supplementary Table S2** and **Supplementary Figure S2**).

### Correlations Between GFC Values and Clinical Variables in Patients With OCD

No correlations were observed between abnormal GFC values and Y-BOCS, HAMD, and HAMA scores in the OCD group with Pearson and Spearman correlation analyses. These correlations were also not significant using the mean FD values and age as covariates with partial correlation analysis.

### ROC Analysis in Patients With OCD and HCs

**Figure 3:**
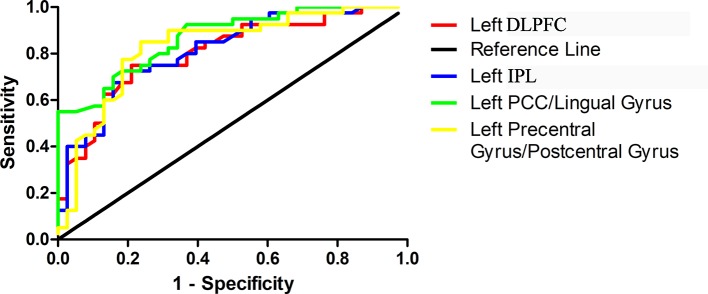
Receiver operating characteristic (ROC) curves using the mean GFC values in the left PCC/lingual gyrus, left precentral gyrus/postcentral gyrus, left DLPFC and left IPL to separate patients with OCD from healthy controls. GFC, global-brain functional connectivity; PCC, posterior cingulate cortex; DLPFC, dorsal lateral prefrontal cortex; IPL, inferior parietal lobule.

ROC curves were conducted with false positive rate (1-specificity) as X-axis and true-positive rate (sensitivity) as Y-axis and were created by connecting all the points on the graph (each cut-off level generated a point) (**Figure 3**). Area under the curve (AUC) is an assessment of the entire diagnostic accuracy of the test, and was computed with SPSS 20.0 software (SPSS Inc., Chicago, Illinois, USA). The AUC values of the left PCC/lingual gyrus, left precentral gyrus/postcentral gyrus, left DLPFC, and left IPL were 0.868, 0.830, 0.804, and 0.814, respectively (**Table 2**).

**Table 2:** ROC analysis for differentiating the patients from the controls by using the GFC values.

Brain regions	Area under the curve	Cut-off point	Sensitivity	Specificity
Left PCC/Lingual Gyrus	0.868	0.3776	72.50% (29/40)	84.21% (32/38)
Left Precentral Gyrus/Postcentral Gyrus	0.830	0.0664	85.00% (34/40)	76.32% (29/38)
Left DLPFC	0.804	0.2552	75.00% (30/40)	78.95% (30/38)
Left IPL	0.814	0.2725	72.50% (29/40)	81.58% (31/38)

ROC, receiver operating characteristic curve; GFC, global-brain functional connectivity; PCC, posterior cingulate cortex; DLPFC, dorsal lateral prefrontal cortex; IPL, inferior parietal lobule.

The results manifested that the GFC value of the left PCC/lingual gyrus could differentiate patients with OCD from HCs with a sensitivity of 72.50% and a specificity of 84.21%. The GFC value of the left precentral gyrus/postcentral gyrus could differentiate patients with OCD from HCs with a sensitivity of 85.00% and a specificity of 76.32%. The GFC value of the left DLPFC could differentiate patients with OCD from HCs with a sensitivity of 75.00% and a specificity of 78.95%. The GFC value of the left IPL could differentiate patients with OCD from HCs with a sensitivity of 72.50% and a specificity of 81.58% (**Table 2**).

The global balanced accuracies of the left PCC/lingual gyrus, left precentral gyrus/postcentral gyrus, left DLPFC and left IPL were 0.7815 (*p* < 0.001), 0.7797 (*p* < 0.001), 0.6826 (*p* < 0.001), 0.6932 (*p* < 0.001) respectively for classifying patients with OCD from HCs using the permutation tests.

## Discussion

In this study, we investigated the GFC alterations in drug-naive patients with OCD with an unbiased method at rest. The primary results showed that patients with OCD exhibited decreased GFC in the region within the DMN (left PCC/lingual gyrus) and increased GFC in the regions within the ECN (left DLPFC and left IPL), which were consistent with our hypothesis. Moreover, ROC analyses indicated that decreased GFC in the left PCC/lingual gyrus and increased GFC in the left DLPFC and left IPL might be used as candidate neuroimaging markers to differentiate patients with OCD from HCs. Inconsistent with our hypothesis, we found decreased GFC in the region within the sensorimotor network, including the left precentral gyrus/postcentral gyrus, and no signiﬁcant relationship was observed between abnormal GFC and clinical variables in the OCD group.

Many previous studies have evaluated FC based on assumptive seed definitions with an ROI method, which may acquire different result patterns. Diverse studies typically generate inconsistent results (2). Furthermore, some studies have focused on the networks of interest and ignored the most crucial brain areas involved in the core pathological abnormalities in OCD (12, 13, 15, 28–30). By contrast, in the present research, we used a voxel-wise brain-wide method to focus on FC alterations in drug-naive patients with OCD at rest. Therefore, the present findings were revealed in an unbiased manner and may detect the potential FC abnormalities involved in the core pathological abnormalities of OCD.

The present study found altered GFC in the left PCC/lingual gyrus, left DLPFC, and left IPL, which indicated that there were disrupted functional interactions within the DMN and ECN in patients with OCD. As a neural substrate for human awareness, the PCC/lingual gyrus regulates arousal and attention, and is a crucial brain region in the DMN (31, 32). Reduced FC in the PCC within the DMN was found in previous studies in patients with OCD (28, 29, 33). Reduced negative correlations between the PCC and the fronto-parietal network were discovered in patients with OCD (33). DLPFC and IPL are the core brain regions in the ECN, which are involved in cognitive task switching, response inhibition, and executive planning (34, 35). In our previous study, high regional homogeneity in the DLPFC and IPL (peaking in angular gyrus) and increased FC between DLPFC and IPL at rest in OCD were found with a different sample (12). Decreased GFC in the DMN and increased GFC in the ECN at rest may be related to low self-awareness and focus on controlling external stimuli in patients with OCD. Furthermore, altered GFC in the DMN and ECN may break the balance of the “triple network” model and leads to the difficulty on switching between task-negative and task-positive processing in patients with OCD (8). In conclusion, our findings suggested that disrupted GFC in the DMN and ECN might contribute to the pathophysiology of OCD.

We also observed decreased GFC in regions within the sensorimotor network (i.e., left precentral gyrus/postcentral gyrus), which indicated that altered GFC within the sensorimotor network might be associated with OCD. Patients with OCD have damaged sensory gating and sensory-motor integration, which demonstrate that the deviance of the sensorimotor network may be involved in the inability of patients with OCD to suppress internally repetitive and intrusive thoughts and behavior (36, 37). Meanwhile, the activation in brain regions within the sensorimotor network in the inhibitory control processes may explain the essence of inhibitory control deficits of OCD (38). Furthermore, a recent study revealed that the amplitude of low-frequency ﬂuctuations of the precentral gyrus showed a large discriminative power to identify patients with OCD from HCs (39).

The diagnostic accuracy is high when AUC is above 0.9, medium when AUC is 0.7–0.9, and low when AUC is 0.5–0.7. On this basis, the accuracies of altered GFC values in the left PCC/lingual gyrus, left precentral gyrus/postcentral gyrus, left DLPFC, and left IPL for differentiating patients with OCD from HCs are moderate. Moreover, the sensitivity and specificity of altered GFC values in these brain regions are relatively high. The ROC results are also validated by permutation tests. Therefore, the ROC results of the present study indicate that the decreased GFC in the left PCC/lingual gyrus and left precentral gyrus/postcentral gyrus and increased GFC in the left DLPFC and left IPL may be used as candidate neuroimaging markers for patients with OCD.

Previous studies have reported correlations between abnormal FC and clinical variables, such as obsessive-compulsive severity and illness duration in patients with OCD (2). However, no relationships were found between abnormal GFC and clinical variables in the OCD group in the current study, which indicated that abnormal GFC values might be trait changes independent of clinical variables for patients with OCD (40).

Surprisingly, we did not obtain abnormal GFC values in the regions within the SN. As an important member in the triple network model, the SN filters and detects the external and internal salient information, and has an important role in supervising the interactions between the DMN and ECN (8). In our previous study, decreased FC strength within the SN, between the SN and ECN, and the DMN in OCD at rest was revealed (15). In that study, we only focused on the SN and ignored other important brain regions involved in OCD. However, in the present study, we explored the entire brain FC with an unbiased voxel-wise method, and the present results were related with the core pathological alterations of OCD.

Global brain connectivity (GBC) has been used to study OCD in a few studies. For example, Anticevic et al. found decreased GBC in the left lateral prefrontal cortex and increased GBC in the right putamen and left cerebellar cortex (41). Moreover, weighted degree centrality (DC), a very similar measure to GBC, has been utilized in some studies. Beucke et al. demonstrated higher DC values in the orbitofrontal cortex and basal ganglia (42). Tian et al. discovered increased DC values distributed in the cortico–striato–thalamo-cortical (CSTC) circuits and parietal, occipital, temporal, and cerebellar regions (43). Shin et al. found that changes of DC value in the right ventral frontal cortex were correlated with improvement of obsessive-compulsive symptoms in patients with OCD after SSRIs treatment (16). Gottlich et al. reported that the lower DC value in the bilateral superficial amygdala could predict treatment outcome of cognitive-behavior therapy in OCD (44). The diverse findings may be due to differences across studies such as global signal regression, head motion, and medication use (2, 45).

Another interesting finding in the current study is that all brain regions with altered GFC values are in the left hemisphere. Brain lateralization is implied in the pathogenesis of psychiatric disorders including OCD (46). Previous studies have found that regional alterations of white matter, gray matter volume, and cortical thickness are mainly located in the left hemisphere, including the middle frontal gyrus, IPL, and precentral gyrus, which may be the foundation for GFC abnormalities in the left hemisphere in patients with OCD (47–49). Combining with previous studies and our results, we inferred that GFC abnormalities in the left hemisphere might contribute to the core pathophysiology of OCD. However, several studies have discovered that the changes of structural and functional asymmetry in patients with OCD are mostly distributed to the right hemisphere (50–52). Sample selection including age, sex, illness duration, medication use, comorbidities, and data analysis, including ROI locations, may account for this discrepancy (2).

This study has several limitations apart from the relatively small sample size. First, we did not collect fMRI data with specific tasks, and abnormal GFC at rest might reveal the general pathological changes in OCD. Second, white and gray matter alterations that may underlie the mechanism of GFC are not evaluated in our research. Thirdly, OCD samples were not divided into different subtypes based on different clinical symptoms due to the sample characteristics (i.e., most patients (32/40) are mixed with both obsession and compulsion) and relatively small sample size. Different OCD subtypes may have different GFC patterns at rest (53). Future studies should consider these issues. Finally, the ROIs were first used as differing in OCD vs HCs, and then used in the ROC analysis, which may lead to double dipping. Therefore, the present candidate neuroimaging markers for OCD should be interpreted cautiously.

Despite these limitations, the current study is the first to evaluate voxel-wise brain-wide FC in OCD in an unbiased manner. The results manifest that abnormal FC patterns of the DMN, ECN, and sensorimotor network may account for the pathophysiology of OCD. The current study provides new insights into the alterations in brain organization function in OCD.

## Data Availability Statement

All datasets generated for this study are included in the article/**Supplementary Material**.

## Ethics Statement

The studies involving human participants were reviewed and approved by the Research Ethics Committee at Qiqihar Medical University, China. The patients/participants provided their written informed consent to participate in this study. Written informed consent was obtained from the individual(s) for the publication of any potentially identifiable images or data included in this article.

## Author Contributions

PL and WG designed the experiments. GC and YO wrote the manuscript. YC, DL, CJ, ZZ, YW, XM, HC, CL, and ZS performed the experiments. WG, YO, and RY analyzed the data. PL, WG, and XW revised the manuscript. All authors contributed to and have approved the final manuscript.

## Funding

This work was supported by grants from Heilongjiang Natural Science Foundation of China (LH2019H064), the Project of Qiqihar Academy of Medical Sciences, China (QMSI2017B-08), the National Key R&D Program of China (2016YFC1307100), the National Natural Science Foundation of China (81771447), Henan Natural Science Foundation Grant (182300410317), and Henan basic and frontier technology research project Foundation Grant (152300410121).

## Conflict of Interest

The authors declare that the research was conducted in the absence of any commercial or ﬁnancial relationships that could be construed as a potential conﬂict of interest.
