# Supplementary material for: Altered Global Brain Functional Connectivity in Drug-Naive Patients With Obsessive-Compulsive Disorder
Source: Front Psychiatry. 2020 Mar 3;11:98. doi: 10.3389/fpsyt.2020.00098 (PMC7062961; doi:10.3389/fpsyt.2020.00098)
Supplement: Supplementary file 1 [file DataSheet_1.doc]

**SUPPLEMENTARY TABLE S1.**

**Demographic and clinical characteristics of participants**

|  | **OCD patients**  **(n =40)** | **HCs**  **(n = 38)** | ***Χ^2^*/*t*** | ***p*** |
| --- | --- | --- | --- | --- |
| Age (years) | 27.28 ± 8.16 | 27.18 ± 8.33 | 0.05 | 0.71 |
| Sex (male/female) | 27/13 | 25/13 | 0.32 | 1.00 |
| Education (years) | 13.40 ± 2.87 | 13.74 ± 3.03 | -0.50 | 0.83 |
| Illness duration (months) | 66.68 ± 75.54 |  |  |  |
| Y-BOCS total score | 24.90 ± 5.73 | 1.13 ± 0.88 | 25.27 | *<0.001* |
| Y-BOCS obsessive thinking | 12.85 ± 4.25 | 0.37 ± 0.49 | 17.98 | *<0.001* |
| Y-BOCS compulsive behavior | 12.05 ± 4.62 | 0.74 ± 0.72 | 14.92 | *<0.001* |
| HAMD | 8.05 ± 4.40 | 1.45 ± 0.95 | 9.04 | *<0.001* |
| HAMA | 10.83 ± 6.55 | 1.16 ± 1.00 | 9.00 | *<0.001* |
| FD | 0.04 ± 0.02 | 0.03 ± 0.01 | 1.25 | 0.13 |
| Time points scrubbed out | 1.13 ± 2.256 | 1.00 ± 2.418 | 0.25 | 0.95 |

OCD = obsessive-compulsive disorder; Y-BOCS = Yale-Brown Obsessive-Compulsive Scale; HAMD = 17-item Hamilton Depression Rating Scale; HAMA = Hamilton Anxiety Rating Scale; FD = framewise displacement.

**SUPPLEMENTARY TABLE S2.**

Regions with abnormal GFC in the patients with OCD with global signal removal

| Cluster location | Peak (MNI) | | | Number of voxels | T value |
| --- | --- | --- | --- | --- | --- |
|  | x | y | z |  |  |
| Left PCC/Lingual Gyrus | -18 | -66 | 12 | 87 | -6.0970 |
| Left Precentral Gyrus/Postcentral Gyrus | -45 | -9 | 30 | 139 | -5.7503 |
| Left DLPFC | -36 | 45 | 27 | 60 | 5.6003 |
| Left IPL | -54 | -42 | 39 | 86 | 4.4181 |

GFC = global-brain functional connectivity; OCD = obsessive-compulsive disorder; MNI = Montreal Neurological Institute; PCC = posterior cingulate cortex; DLPFC = dorsal lateral prefrontal cortex; IPL = inferior parietal lobule.


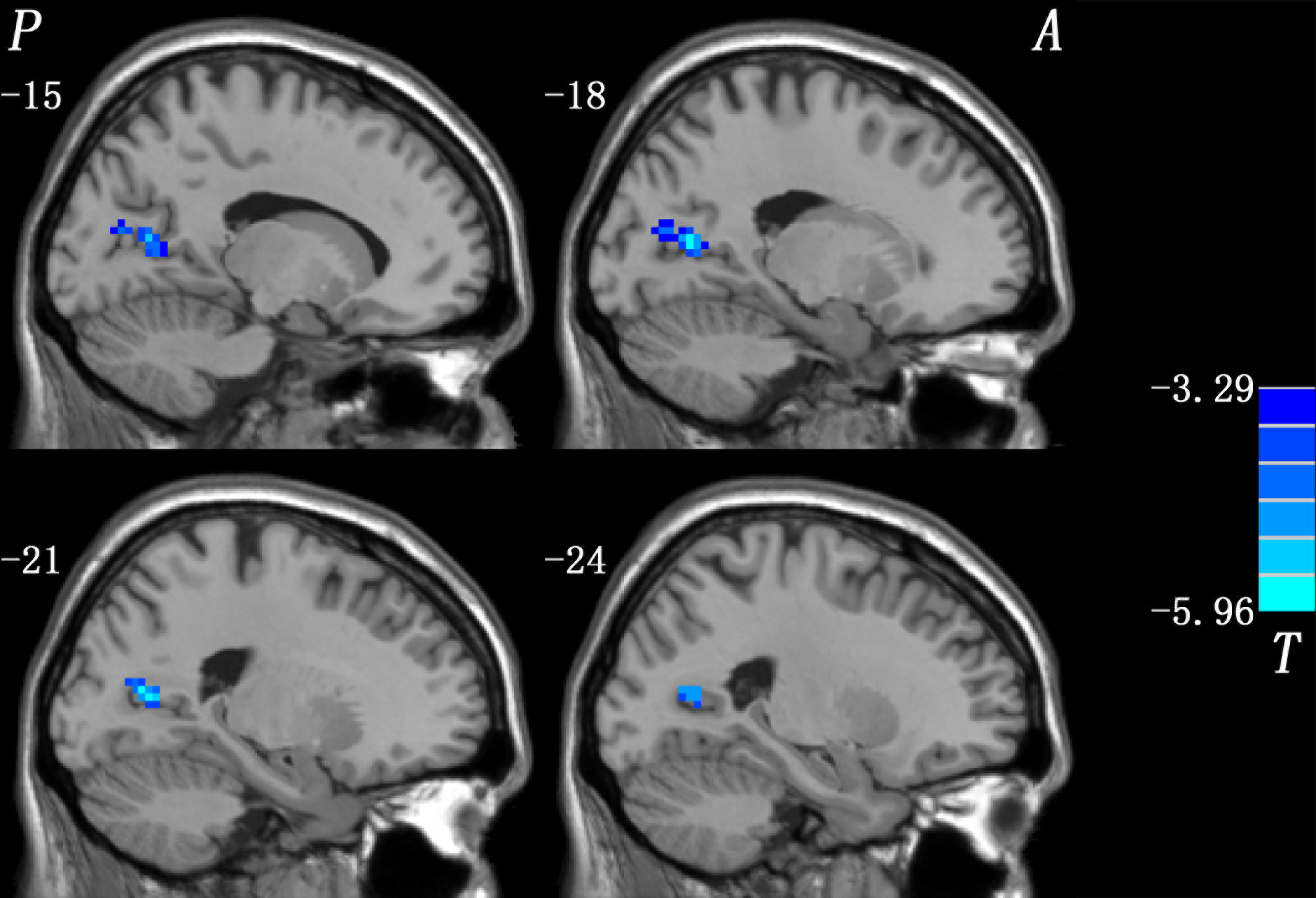


**Figure S1.** A sagittal view of the left PCC/lingual gyrus cluster. The threshold was set at *p* < 0.05 corrected by the GRF method. Blue denotes decreased GFC. Colour bar indicates *t* values from two-sample *t*-tests. PCC = posterior cingulate cortex; A = anterior side; P =posterior side; GRF = Gaussian Random Field; GFC = global-brain functional connectivity.


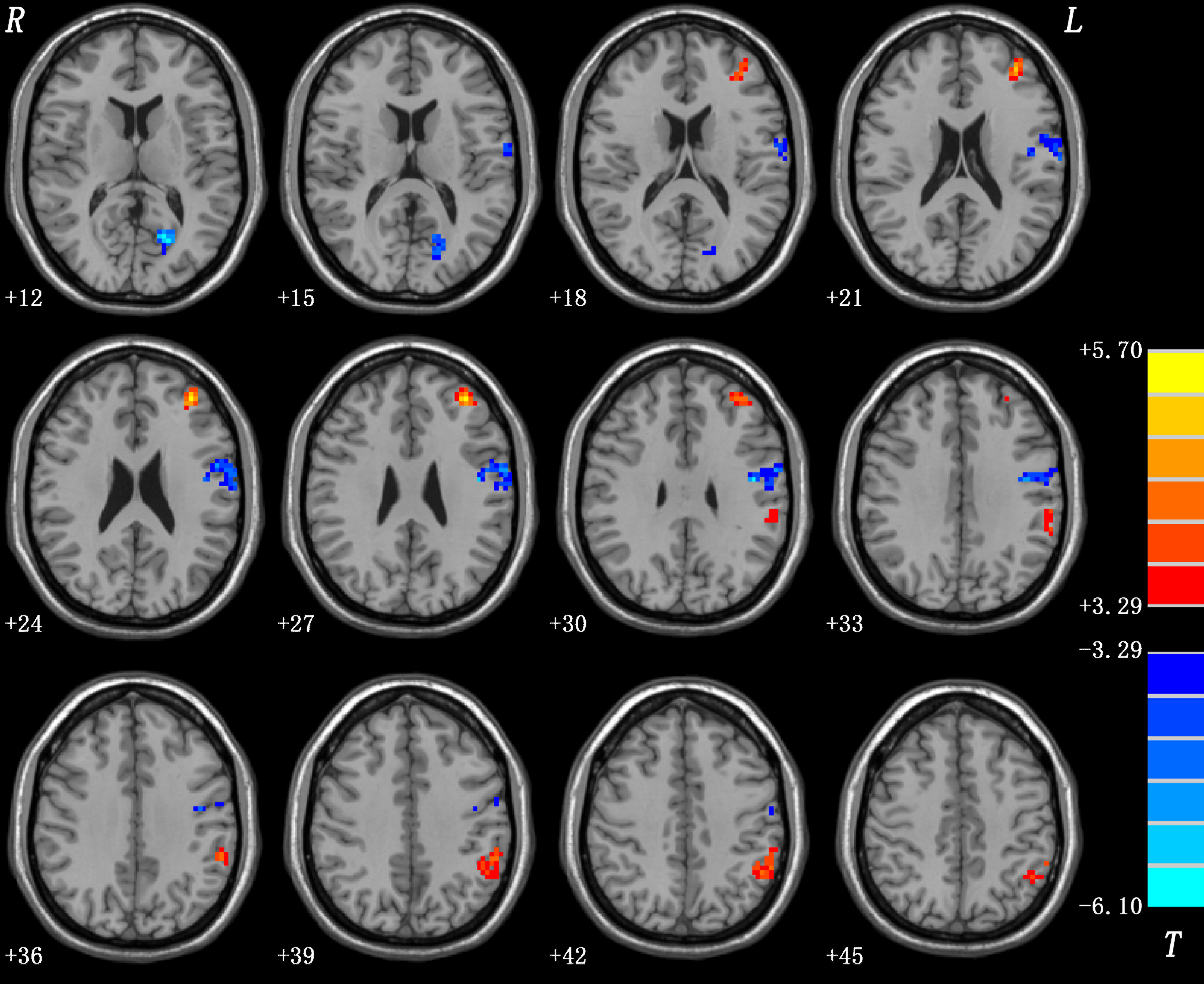


**Figure S2.** Brain regions with abnormal GFC values in patients with OCD with global signal removal. The threshold was set at *p* < 0.05 corrected by the GRF method. Red and blue denote increased and decreased GFC values respectively. Colour bar indicates *t* values from two-sample *t*-tests. L = left side; R = right side; GFC = global-brain functional connectivity; OCD = obsessive-compulsive disorder; GRF = Gaussian Random Field.
